# Letter to the editor on ‘electrical flash burns due to switchboard explosion’

**DOI:** 10.1111/iwj.14038

**Published:** 2022-11-25

**Authors:** Li Zhang, Chun‐Xia Hou, Hai‐Hong Li

**Affiliations:** ^1^ Department of Pharmacy, Changzhi People's Hospital Changzhi Medical College Changzhi China; ^2^ Drug Clinical Trial Institution, Changzhi People's Hospital Changzhi Medical College Changzhi China

Dear Editor,

We recently read with great interest by Chao Lian et al.^1^ about ‘Electrical flash burns due to switchboard explosion’. Electrical injuries are relatively uncommon in clinical practice. Although approximately 27% of admissions to burns units in developing countries are due to electrical injuries, the consequences can be devastated.^2^ Electrical injuries mainly occur in young males who work in the industrial workplace and is the fourth main reason of traumatic occupational death.^3^ Facing such patients with severe electrical flash burns, modified moist occlusive burn therapy (MMOBT) may be an alternative treatment, but there are some shortcomings from our perspective.

Firstly, the number of cases in this study was relatively small, which could reduce the credibility of the article. It is expected that the authors could increase the sample size in future studies.

Secondly, the novel therapy utilised in this study is called MMOBT which is achieved by the combination application of chitosan‐based biogel and a layer of sterile polyethylene film. If the authors would like to proceed an in‐depth study, some typical medicines could be compared such as the Moist Exposed Burn Ointment (MEBO): MEBO is a Chinese burn ointment which was registered USA patented formulation in 1995.^4^ MEBO is a good topical agent for the treatment of burn injuries due to its ease of patient movement and fine healing properties.^5^

Finally, it is well‐known that the consequences of electrical injuries can be destructive. However, we prefer to know more about the following points: First, what is the conceptual distinction between electrical injury and electrical flash burn? Second, what is the difference between the consequences of high voltage current and low voltage current? Third, what is the condition for the occurrence? It is hoped that you could give us more details regarding questions mentioned above.

In conclusion, this study indicated that MMOBT may be an effective therapeutic strategy for the treatment of electrical flash burns. We are looking forward to the further research in near future.

## AUTHOR CONTRIBUTIONS

Li Zhang wrote the manuscript and handled the submission process. Chun‐Xia Hou and Hai‐Hong Li were involved in literature review and revision of the first draft.

## CONFLICT OF INTEREST

The author(s) declared no potential conflicts of interest with respect to the research, authorship, and/or publication of this article.

## Data Availability

Data sharing not applicable to this article as no datasets were generated or analysed during the current study.
